# Supplementary material for: Association between oral microbiome and seven types of cancers in East Asian population: a two-sample Mendelian randomization analysis
Source: Front Mol Biosci. 2023 Nov 21;10:1327893. doi: 10.3389/fmolb.2023.1327893 (PMC10702768; doi:10.3389/fmolb.2023.1327893)
Supplement: Supplementary file 1 [file DataSheet1.ZIP › Supplementary Materials/MR plots for tongue/tongue═╝/Prostate cancer/pheno.3250_to_prostate cancer_leave_one_out.pdf]

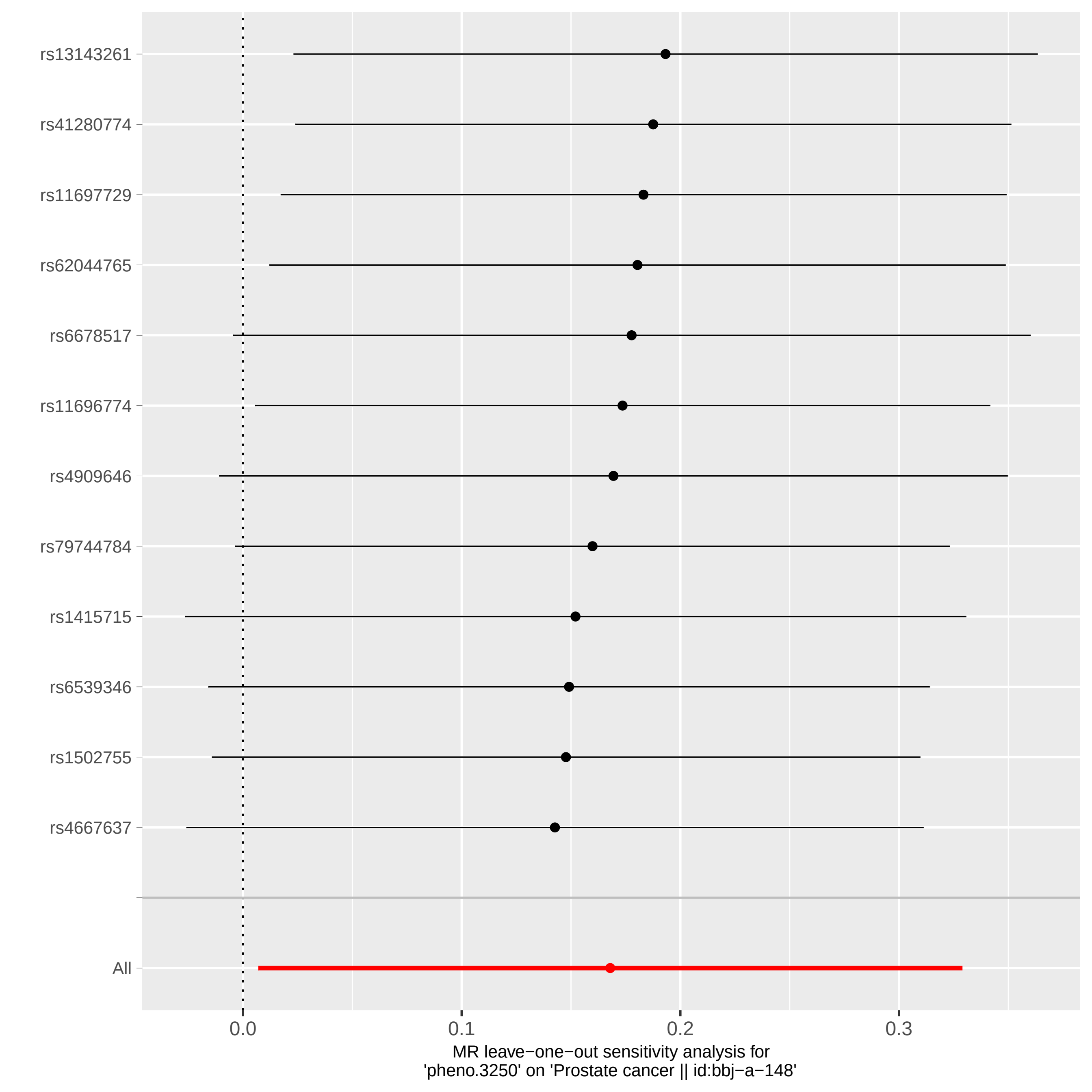
rs13143261
rs41280774
rs11697729
rs62044765
rs6678517
rs11696774
rs4909646
rs79744784
rs1415715
rs6539346
rs1502755
rs4667637
All
0.0
0.1
0.2
0.3
MR leave−one−out sensitivity analysis for
'pheno.3250' on 'Prostate cancer || id:bbj−a−148'
